# An operating room employee with a necrotic fingertip

**DOI:** 10.1099/jmmcr.0.005138

**Published:** 2018-01-22

**Authors:** Janna S. E. Ottenhoff, Geert P. Voorn, Bart J. M. Vlaminckx, Philip G. Juten, Gertjan H. J. Wagenvoort

**Affiliations:** ^1^​ Department of Plastic Surgery, St. Antonius Hospital, P.O. Box 2500, 3430 EM Nieuwegein, The Netherlands; ^2^​ Department of Medical Microbiology and Immunology, St. Antonius Hospital, Nieuwegein, The Netherlands

**Keywords:** needlestick injury, Streptococcus group A, mycotic aneurysm, lymphangitis, bacterial infection, intravenous benzylpenicillin

## Case summary

A 50-year-old, male, operating room employee presented with a swollen and painful left index finger and progressive lymphangitis on his left arm (patient A). Despite self-usage of antipyretics (paracetamol) and amoxicillin/clavulanate, prescribed by his general practitioner 1 day before presentation, he developed rigors.

The symptoms started 2 days after assisting an open repair of a ruptured abdominal aortic aneurysm in a 73-year-old male (patient B).

**Fig. 1.**
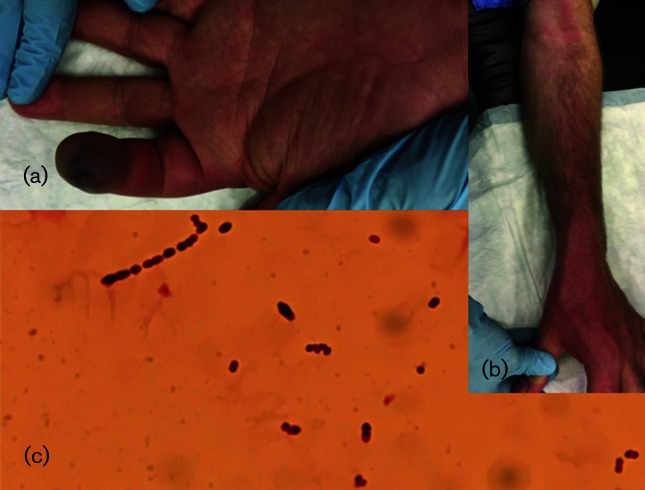
(a) Patient A: swollen and necrotic left index fingertip; (b) Patient A: lymphangitis of the left arm; (c) Patient B: Gram-stain-positive cocci in chains.

Vital parameters of patient A at first presentation were within the normal range: temperature 37.9 °C, blood pressure 121/78 mm Hg and pulse rate 90 bpm. The results for laboratory tests performed on admission were notable for a white blood cell (WBC) count of 18.4×10^9^ cells l^−1^ (reference, 2.5–8.2×10^9^ cells l^−1^) with 90.9 % neutrophils, and a CRP of 86 mg l^−1^ (reference, <10 mg l^−1^). Physical examination revealed a swollen and necrotic fingertip of the left index finger (Fig. 1a). Flexion was limited, and palpation of the tendon at the mid phalanx was painful. Examination of the left arm revealed lymphangitis on the dorsoradial side of the under- and upper arm, reaching up to the left axilla (Fig. 1b). Intravenous treatment with benzylpenicillin and clindamycin was initiated. Exploration of the index finger by incision revealed transparent serous fluid but no pus. A sample was obtained for Gram staining and culture.

In patient B, Gram staining of the aortic thrombus revealed Gram-stain-positive cocci in chains, confirming the diagnosis of a mycotic aneurysm. Cultures obtained from blood and the mycotic thrombus were found to be positive (Fig. 1c).

**Question**

What was the underlying cause of infection and lymphangitis of patient A?

**Answer options**

1. Erysipeloid infection following needlestick-injury-mediated transmission of *Erysipelotrix rhusiopathiae.*

2. Sporotrichosis (*Sporothrix schenckii* infection) following a rose prick injury while gardening.

3. Needlestick-injury-mediated transmission of *Streptococcus pyogenes.*

4. Transmission of *Staphylococcus aureus* owing to contamination in the operating room.

## Discussion

**Correct Answer:** 3. Needlestick-injury-mediated transmission of *Streptococcus pyogenes*.

The symptoms of patient A started 2 days after a needlestick injury while assisting during an operation. The needle, which hit his left index finger, had been used earlier for stitching the mycotic aneurysm of patient B.

Cultures obtained from blood and from the mycotic thrombus of the aneurysm in patient B revealed *Streptococus pyogenes,* which was also isolated from the wound cultures of patient A. The group A streptococcal (GAS) strains of patients A and B were indistinguishable in antibiotic susceptibility pattern (penicillin-, erythromycin-, clindamycin- and trimethoprim-susceptible and doxycycline-resistant), emm-type (emm 8.3 – rarely found) and molecular genetic typing, using amplification fragment length polymorphism (AFLP) analysis. These findings suggest a rarely described case of needlestick-injury-mediated transmission of invasive group A streptococcal disease.

After intravenous treatment with benzylpenicillin and clindamycin, the physical signs and lab values of patient A progressively improved. The wound of his index finger, however, remained necrotic and was left for demarcation to heal per secundam. Patient A was discharged with amoxicillin prescribed orally for an additional week. Follow-up after 3 weeks revealed a dry, necrotic plaque without signs of infection. Patient B was successfully treated with a 6-week course of intravenous benzylpenicillin.

Transmission of GAS strains has caused death in many women owing to puerperal sepsis. High mortality rates after childbirth owing to puerperal sepsis decreased rapidly when Semmelweis instituted hand washing in his obstetric clinic in 1847 [1]. Nowadays, GAS still causes severe invasive infections, which can progress rapidly and requires aggressive therapy. A recent review of the literature shows fatality rates of 15 % in cases with invasive GAS infection [2].

Transmission of GAS strains to healthcare workers has been described following cardiopulmonary resuscitation and following necropsy [3, 4]. The transmission of GAS strains following needlestick injury is rare and only two previous cases have been published. The first case describes GAS-transmission that resulted in necrotizing fasciitis after inserting a central venous catheter in a septic patient [5]. The second documented case is similar to the case described here: a surgeon developed rigors and lymphangitis within 36 hours after injury by a medical instrument used to treat a patient with GAS infection [6]. These cases illustrate the potential risk of GAS transmission and serious infections associated with needlestick injuries in healthcare workers. The risks of HIV, B and hepatitis C transmission are well recognized, as opposed to the risk of GAS transmission. Healthcare workers should be aware of this risk after blood-contaminated injuries involving an index patient with GAS infection. Rapid recognition and aggressive treatment of invasive GAS infection is of great importance to prevent development of this severe disease.
